# Infant nutrition in the first seven days of life in rural northern Ghana

**DOI:** 10.1186/1471-2393-12-76

**Published:** 2012-08-02

**Authors:** Raymond Akawire Aborigo, Cheryl A Moyer, Sarah Rominski, Philip Adongo, John Williams, Gideon Logonia, Gideon Affah, Abraham Hodgson, Cyril Engmann

**Affiliations:** 1 Navrongo Health Research Centre, Post Office Box 114, Navrongo, Ghana; 2 University of Michigan, Ann Arbor, Michigan, USA; 3 University of Ghana, Legon, Greater Accra Region, Ghana; 4 University of North Carolina, North Carolina, USA

**Keywords:** Infant feeding, Breastfeeding, Early neonatal, Neonatal

## Abstract

**Background:**

Good nutrition is essential for increasing survival rates of infants. This study explored infant feeding practices in a resource-poor setting and assessed implications for future interventions focused on improving newborn health.

**Methods:**

The study took place in the Kassena-Nankana District of the Upper East Region of northern Ghana. In-depth interviews were conducted with 35 women with newborn infants, 8 traditional birth attendants and local healers, and 16 community leaders. An additional 18 focus group discussions were conducted with household heads, compound heads and grandmothers. All interviews and discussions were audio taped, transcribed verbatim and analyzed using NVivo 9.0.

**Results:**

Community members are knowledgeable about the importance of breastfeeding, and most women with newborn infants do attempt to breastfeed. However, data suggest that traditional practices related to breastfeeding and infant nutrition continue, despite knowledge of clinical guidelines. Such traditional practices include feeding newborn infants water, gripe water, local herbs, or traditionally meaningful foods such as water mixed with the flour of guinea corn (yara’na). In this region in Ghana, there are significant cultural traditions associated with breastfeeding. For example, colostrum from first-time mothers is often tested for bitterness by putting ants in it – a process that leads to a delay in initiating breastfeeding. Our data also indicate that grandmothers – typically the mother-in-laws – wield enormous power in these communities, and their desires significantly influence breastfeeding initiation, exclusivity, and maintenance.

**Conclusion:**

Prelacteal feeding is still common in rural Ghana despite demonstrating high knowledge of appropriate feeding practices. Future interventions that focus on grandmothers and religious leaders are likely to prove valuable in changing community attitudes, beliefs, and practices with regard to infant nutrition.

## Background

Infant and young child nutrition is a vital component of childhood care and a major determinant of short- and long-term health outcomes in children [1]. Good infant nutrition stimulates intellectual development and is also associated with improved infant health, stronger immune systems, lower risk of non-communicable diseases(such asdiabetes and cardiovascular disease), and longevity [2]. Protecting, promoting and supporting infant and young child feeding is therefore essential for the healthy growth and development of children [3].

For newborn infants, the World Health Organization (WHO) and United Nations Children’s Fund (UNICEF) endorse breastfeeding as an integral part of the reproductive process, the natural and ideal way of providing complete nutrition, and a process that provides a unique biological and emotional basis for child development [4]. The benefits of breast milk, which include reduced risk of diarrhea, pneumonia, otitis media and other infections, have been well established [5,6] and the strategies to guide mothers to breastfeed appropriately are available [3,7].

As a public health measure, the Global Strategy for Infant and Young Child Feeding jointly developed by WHO and UNICEF recommend initiation of breastfeeding within an hour of birth and exclusive breastfeeding for six months for all infants [7,8].This position was reaffirmed in 2011 [9]. For a child to be exclusively breastfed, there should be no prelacteal intake of anything solid or liquid other than breast milk, medications or vitamins. Giving a child any amount of water, gripe water, juice or porridge is not considered “exclusive breastfeeding.” This recommendation is worldwide, applying to infants of mothers in low- as well as high-income countries.

Breastfeeding should be initiated within an hour of birth and exclusively provided for the first six months of life [10].The introduction of supplementary feeding before the age of six months in low-income countries has been associated with increased morbidity and mortality, especially from diarrhea and acute respiratory infections [10,11]. In a recent Cochrane review, the authors concluded that based on the results of two controlled trials and 18 other studies, exclusive breastfeeding for six months has several advantages over exclusive breastfeeding for three to four months. The main newborn advantage included a lower risk of gastrointestinal infection, although a reduced level of iron in some low –income settings was observed by some authors. For the mother, the main advantage was more rapid maternal weight loss after birth, and a delayed return of menstrual periods which can contribute to more favorable birth spacing. No reduced risks of other infections or of allergic diseases were reported [12].

Breast milk is widely available, economical, and sterile, yet many infants are not breastfed according to the recommendations [13,14]. Lauer and colleagues estimate exclusive breastfeeding in West Africa for infants under six months to be only 6.1% [15]. In Ghana, West Africa, where infant and neonatal mortality rates are 47 and 29 per 1000 live births respectively, 52% of mothers initiate early breastfeeding (i.e. within an hour of birth) and 63% report exclusively breastfeeding their infants for 6 months [16,17]. A recent study of 10,947 breastfed infants in the Kintampo district in Ghana found a marked dose–response relationship between delayed initiation of breastfeeding (one hour to seven days), and neonatal death, with an overall 2.4 fold increase in neonatal death with initiation later than one day [18].The authors concluded that all-cause neonatal mortality could be reduced by 16.3% if all infants initiated exclusive breastfeeding on day 1 of life and by 22.3% if initiation took place within the first hour.

Improving initiation and duration rates of breastfeeding represent a unique opportunity to modify neonatal and under–five mortality rates to achieve Millennium Development Goal 4. Despite a relatively high percentage of “exclusive breastfeeding” reported in Ghana [16,17], anecdotal experience suggests there may be regional differences. In addition, exactly what mothers feed their infants aside from breastmilk in rural Ghana is not well understood. The current study sought to answer the following research questions in one region in rural northern Ghana: 1) What do mothers with newborn infants report with regard to breastfeeding initiation and supplementation? and 2) Are there cultural practices that influence what infants are given in the first week of life in this region of Ghana? In exploring the answers to those research questions, we also aimed to address potential implications of our findings with regard to future interventions to improve the health of infants in rural northern Ghana.

## Methods

### Research site

The research was carried out in the Kassena-Nankana District (KND). The predominantly rural district has a population of 151,955 [16]. Two main ethnic groups in the district, the Kassenas and the Nankanis, share one hospital located in the administrative capital, Navrongo. In addition, five community health centres in the district provide static health services to the communities. Subsistence agriculture is predominant, and poverty is widespread. There is little electricity, few health facilities and many transportation challenges, all of which are representative of rural West Africa. The district is home to the Navrongo Health Research Centre (NHRC), which has been carrying out research in the area for more than 20 years.

### Sampling

The Navrongo Health Demographic Surveillance System (NHDSS) that operates within the NHRC has demarcated the Kassena-Nankana District into five zones to facilitate the monitoring of demographic dynamics. The North and West zones are inhabited predominantly by Kassenas while the East and South are mostly Nankanis. In order to elicit ethnic differences in infant feeding practices if any, ethnicity was considered in the selection of participants and interviewees. For ease of data collection, one zone was randomly selected – the North (Kasem) and South (Nankani) - for each of the ethnic groups. The Central zone, which hosts heterogeneous ethnic groups, was not included in the sample frame.

The targets of this research included women with newborn infants (including those who had delivered at home and in facilities), grandmothers, compound heads, household heads, community leaders, formal health care providers and traditional health care providers. In keeping with exploratory qualitative methodology, the sampling strategy was developed to maximize the diversity of respondents selected and as such no formal sample size calculations were conducted. “Women with newborn infants” were defined as women who had delivered an infant more than 4 weeks prior but not longer than 8 weeks. This time frame was chosen to maximize their ability to remember details of the delivery experience. “Grandmothers” were defined as any woman who had at least one grandchild born within the previous year. “Formal health care providers” included physicians, nurses, midwives, and medical assistants. “Traditional health care providers” included traditional birth attendants, herbalists, and other local healers not recognized by the medical establishment. In addition to women with newborn infants, a second group of mothers with newborns who were 7 days old (early neonatal period) were also interviewed.

Community Key Informants (CKIs),who work with the Navrongo Health Research Centre to routinely collect information on vital events that occur in their communities including births, deaths, pregnancies and marriages, were contacted for a list of mothers whose infants had reached 1 month of age. The list of mothers was then categorized based on literacy, place of delivery, and number of previous deliveries. These “stratifiers” were chosen to maximize the variability of our sample, assuming that women who delivered in a facility might have different attitudes and beliefs regarding breastfeeding and infant nutrition than women who delivered at home, for example. Similarly, women who have never experienced childbirth before the recent delivery may have different perceptions than women who have had one or more previous deliveries. Within each of those groups, mothers who could be contacted immediately after the child was 29 days old were purposively selected for interview. Given the small and tight knit nature of these communities, minimal demographic information was collected on each respondent to ensure anonymity.

Health care providers working in the district were also interviewed. Eight IDIs were planned with nurses/midwives. Medical assistants sometimes stand in for midwives during deliveries, and thus medical assistants were asked to participate in IDIs as well. Medical doctors are generally found only in hospitals, thus selection of doctors for the IDIs was done at the district hospital. The Senior Medical Officer (SMO) in-charge of the district hospital was purposively selected while the second doctor was conveniently sampled on the day that the interview with the SMO was held.

Traditional Birth Attendants (TBAs), herbalists, and other local healers outside the formal health care system were purposively selected within the selected zones. Their selection was done through the CKIs who identified potential respondents based on the individual’s knowledge and/or involvement with maternal and child health at the community level.

With regard to focus groups, in each selected zone, five community clusters were randomly selected for the purpose of focus group recruitment. CKIs who live in those communities were consulted in identifying grandmothers with relevant experience in neonatal health residing within the selected clusters. Four focus group discussions were conducted with grandmothers. Each FGD included 8 – 10 grandmothers. In addition, a random list of 20 household heads and 20 compound heads from the same community was generated from the NHDSS database for focus group discussions with household heads and compound heads. The individuals were then contacted in the order that they appeared on the list and the first 12 to grant consent were invited to participate in the discussions.

### Data collection

A variety of qualitative research techniques were used, including In-depth Interviews (IDIs) and Focus Group Discussions (FGDs). All IDI and FGD instruments were developed, pretested, and revised to ensure maximum comprehension. All interviews were conducted by trained field staff employed by the Navrongo Health Research Center (NHRC). Interviewers went through at least one week-long interviewer training session led by one of the co-investigators (RA), totaling nearly 25 hours of instruction and mock interviews. All interviewers conducted a pretest interview that was reviewed and discussed to optimize data collection and ensure consistency across the interviewers. Half of the interviewers had been through the training repeatedly, given that they had worked on several NHRC studies in the past.

A total of six individuals conducted the interviews and focus groups for this project. Four were Ghanaian (two were undergraduates, two were graduate students at a nearby university; three were male, one was female) and two were from the United States (both were female medical students). The American interviewers conducted interviews with English-speaking health care providers; the Ghanaian interviewers conducted all remaining interviews. Ghanaian interviewers were fluent in both the respondent’s native language (either Kasem or Nankani) but also in English, the official language in Ghana. Although the interviewers were fluent in the local languages, the interviewers did not come from the communities where the interviews were conducted. There were no known relationships between interviewers and participants.

In-depth interviews (IDIs) were one-on-one interviews relying upon a semi-structured instrument and detailed probes to guide the discussion. The interviews occurred mostly in respondents’ homes and in the health care setting (for the health care workers) and typically lasted between 45 and 60 minutes. All interviews were audio recorded, and notes were kept on verbal and non-verbal communication by a second field team member present at each interview. The interviews were transcribed into English, with unique words and phrases or those that were difficult to translate remaining in the local language. Interviews with health care providers were conducted in English and transcribed verbatim.

Focus groups were conducted with participants typically gathered in a semi-circle around the interviewer. Questions were posed to the group, and the interviewer took responses from participants one by one, moving the hand-held microphone closer to the respondent who was speaking. FGDs typically lasted between 60 and 90 minutes. All focus groups were audio recorded, conducted in the local language, and transcribed into English as described above.

All transcribed data, including field notes, were read by at least three of the co-investigators (RA, CM, CE) and reviewed for completeness and clarity. Any sections of the interviews that were unclear were discussed as a group, including reviewing audio files and discussing the issues with the interviewers.

### Data analysis

Three of the investigators (CE, CM, RA) conducted “in vivo” coding to assist in the identification of main codes. This involved making written notes on hard copies of the transcripts and reviewing the notes together. From the in vivo coding, a preliminary coding structure was agreed upon and a codebook was created. At that point, all transcripts imported were into NVivo 9.0, a qualitative software analysis package. Focused coding (using the initial coding structure as a guide) was conducted by four separate coders, including one of the investigators (CM). The remaining three were master’s trained public health researchers.

As coding progressed, additional codes and subcodes were added to the codebook and the information gleaned was fed back to the interviewers in the field. As a result, interviews in the latter half of the data collection period focused more tightly on the early neonatal period. This was in response to perceived saturation of the prenatal codes and yet insufficient information about exactly what happens at the time of delivery.

Coders held regular coding meetings during which time the meanings of any code that came into question were revisited and discussed among the group. The codebook was revised to reflect inclusion and exclusion criteria that may not have arisen previously.

### Ethical considerations

Prior to the commencement of the research study, permission was sought from local leaders to enter into their communities to conduct the study. Permission was also received from the District director of health services and the Senior Medical Officer in-charge of the hospital to carry out the interviews with their staff. Each interviewee or discussant also had the aims, objectives, risks and benefits of the study explained to them. Only individuals who agreed to be interviewed or to participate in a discussion did so. Permission to audio record the interviews and discussions was also sought from the participants.

Ethical approval for the study was sought and obtained from the institutional review boards of the Navrongo Health Research Centre, and the Universities of Michigan and University of North Carolina before initiating the study.

## Results

**Figure 1:**
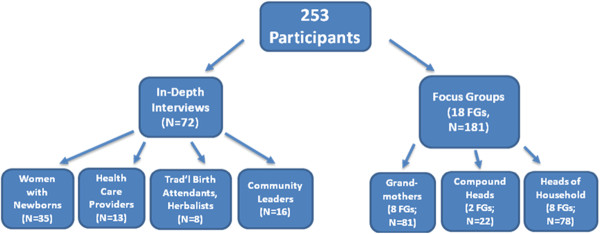
**Participants **[17]**.**

A total of 253 individuals from the Kassena-Nankana district in Ghana participated in either in-depth interviews or focus group discussions between July 1 and November 1, 2010. In-depth Interviews were conducted with 35 women with newborn infants, 13 health care providers, 8 traditional birth attendants / herbalists, and 16 community leaders. In addition, focus group discussions were conducted with 81 grandmothers, 22 compound heads, and 78 heads of households (See Figure 1.)

### Community knowledge of breastfeeding guidelines

Community members demonstrated knowledge of the recommended guidelines for newborn feeding. Most respondents reported being aware that the baby must be put to the breast immediately after delivery, breastfeeding should be on demand and that colostrum ‘*makes the baby healthy and strong…*’ (FGD, Compound Heads). Babies ‘*suck the colostrum because it cleans the dirt inside (the baby) and make him grow healthy’* (FGD, Grandmothers). Community members also report an appreciation for the value of breastmilk: ‘*It is (the) milk that protects the baby against diseases.*’ (FGD, CompoundHeads).

Women with newborn infants and grandmothers in the community reported that nurses at antenatal clinics provided guidance on breastfeeding. Mothers are told ‘*to be allowing (their) babies to suck the first breast milk because it is good for their health’* (FGD, Grandmothers), and that they should ‘*not give water until six months’ time.’* (IDI, mother of new born, one delivery). This was confirmed in the health worker interviews in the dialogue below:

"R: Well I know that these days, they are encouraging them to put the baby straight to the breast as soon as the baby is born."

"M: So seconds?"

"R: Yes probably. Yes, seconds. Well I mean, once a midwife does the delivery, the first thing the midwife would do would be to try to encourage the woman to breastfeed. (IDI, Health Care provider)"

However, awareness of the guidelines does not necessarily ensure appropriate breastfeeding practices. For instance, one grandmother reported that ‘*They have told us that the baby should not drink water until six months but some will ignore that and still give the baby water.’* (FGDGrandmother).

### Initiation and maintenance of breastfeeding

Participants in this study reported high levels of breastfeeding. Further, many respondents mentioned that mothers initiate breastfeeding within 30 to 60 minutes after delivery. Intervals between delivery and breastfeeding were reported to be influenced by place of delivery, sex of the child, availability of breast milk, bitterness of breast milk, and age of the mother. Typically, when a birth takes place in the community, some routines and rituals are performed before the baby is fed. These include cutting of the umbilical cord, bathing of both mother and baby, and washing of the breasts. Some of these routines are based on advice from health care providers.

"The nurses have taught us that immediately (after) the baby is born and you cut the umbilical cord and the baby cries you have to teach the mother to wash her nipples very well. Then you hold the breast into the baby’s mouth. If there is no breast milk and he continues suckling it will come. (FGD, Grandmother)"

"Some are however rooted in tradition. For instance, traditionally, ‘if it is the woman’s first delivery, it can take 3 or 4 days depending on the sex of the baby’ (FGD, Household heads). One mother reported that ‘On the first day, the baby did not breastfeed, the following day the baby did not breastfeed too, it was the third day that I breastfed the baby’ (IDI, Mother of newborn)."

Healthcare providers – both formal and traditional providers – said that women are encouraged to follow breastfeeding guidelines: that the child has to be put to the breast immediately after delivery, fed breastmilk alone for the first six months and only thereafter supplement breastmilk with family foods.

"R: According to the breastfeeding practices in the hospital, exclusive breastfeeding is encouraged so immediately the baby is put to the breast. The baby is given to the mother to hold it and to start breastfeeding. (IDI, Health care provider)"

"M: Do mothers usually try to breast feed their babies after delivery?"

"R: Yes the mother is always trying to let the baby suck the breast because if he refuses to suck it means he is not well."

"M: How soon after delivery?"

"R: Within some minutes"

"M: How many minutes?"

"R: Like five minutes (IDI, Traditional healthcare provider)"

Mothers use varied signals such as the baby crying or anytime the baby sleeps and wakes up to determine whether their babies are hungry and need to be breastfed or not. For instance, once ‘*a baby sleeps and wakes up, it is a must for the baby to breastfeed’* (FGD, Grandmothers). Respondents found it difficult to estimate the frequency of breastfeeding for the first week of life. Those who tried to estimate the frequency of feeding gave figures ranging from 3 to 30 times a day or about 100 to 200 times a week.

Community members also reported practical difficulties in breastfeeding newborns. These range from mothers not knowing how to hold the breast properly to skills required to increase breast milk if the mother’s supply is deemed inadequate. Some mothers are given ‘*local herbal concoctions to drink and some to massage the breast*’ (IDI, Mother of newborn, supervised delivery) to stimulate the production of breast milk. Others are also given *yara’na* (flour mixed with water) to drink.

"I for instance, when I gave birth and I had no breast milk that is what they gave me (herbs that they peel from the back of trees) and I mixed them with millet flour with water and drunk it. When I drunk it, I saw changes in my breast milk; it gave me enough breast milk. (IDI, Mother of new born, 2 or more children)."

Other methods of stimulating the production of breast milk include mixing millet flour with shea-butter and drinking it, massaging the breast with shea-butter and fetching the bark of a thorn tree and cooking it with vegetables for the lactating mother to eat. Also, where the mother cannot breastfeed, ‘*they will look for a nursing mother who is around the community and let this woman breast feed the baby.’*(FGD, Household head).

Respondents noted several factors that contribute to the early cessation of breastfeeding. Respondents indicated that anxiety is common among mothers whose babies do not feed well, which can result in disregarding feeding guidelines. One grandmother reported that although ‘*some women will say they have heard the law (guidelines regarding exclusive breastfeeding), but since she has not got enough breast milk to feed the baby, what will she do; they will still give the water and herbs to supplement the breast milk*’ (FGD, Grandmother). Data suggests that other mothers simply did not want to breastfeed. Younger mothers were reported to be reluctant to breastfeed so that they can keep the shape of their breast. Healthcare providers also observed that some women have cracked and engorged nipples which make breastfeeding very painful and therefore such mothers are not motivated to breastfeed.

### Supplementary feeding

Newborns in the district are fed a wide variety of foods in addition to breastmilk. These include formula, gripe water, warm water, herbal concoctions and water from the flour of guinea corn (*yara’na*). Some mothers view gripe water as medicine for the baby. It is given to the baby in case of a stomachache. Warm water is usually fed to the child and it is meant to create ‘*appetite (for the baby) to be able to suck the mother’s breast milk well’* (FGD, Grandmothers). In cases where infants are not breastfeeding well, local healers often recommend supplemental feeding. As one grandmother described,

"M: What do you normally do if a woman has breastfeeding difficulties?"

"R: Now if a woman has such a problem we send the baby to the healers and they will prescribe food to be bought for the baby to feed.(FGD, grandmother)"

### Cultural practices surrounding breastfeeding

Data suggests that there are several cultural traditions and practices associated with breastfeeding in this region of northern Ghana. Traditionally, first time mothers known as *kacheeri* in Kasem and *sari sari doka* in Nankani, are required to express their first milk into a container and put black ants in it to test for bitterness. If the ants succeed in crawling out, the milk is declared wholesome and the mother can go ahead and breastfeed. On the other hand, if the ants die, the breast milk is considered bitter (*bisitoo* in Nankani and *yili-kweo* in Kasem), dirty and poisonous and can give the child diarrhea, which could lead to death. The mother must therefore go through a rite called *puure-nyoone in* Kasem and *wobi-biisa* in Nankani, to purify the milk before initiating breastfeeding. *Puure-nyoone* or *wobi-biisa* involves the use of herbs or shea-butter to rub or wash the breasts. The duration of *puure-nyoone* or *wobi-biisa* depends on the sex of the child. Generally, it lasts three days for mothers of male babies and four days for mothers of female babies. When a *kacheeri* or a *sari saridoka* has gone through these rites, it is assumed that the breastmilk is no longer bitter and the mother can initiate breastfeeding.

"If it is the first time the woman delivers. They will press out the woman’s breast milk into a container and put some ants inside it. If the ants die, it means the breast milk is bitter and not good for the baby but if the ants don’t die then they will allow it to suck the breast milk. (FGD, Compound heads)"

Also first-time mothers are expected to go through a cultural cleansing known as *sooru* in Kasem and *kosoto* in Nankani, regardless of the bitterness of their breastmilk. The process involves the pouring of warm herbal water over the mother for a period of three days if the child is a male and for four days if the child is female. In some communities, during the period of the cleansing, either a wet nurse is used or the child is fed on herbal teas because the mother is not allowed to breastfeed. While a new mother is going through *sooru* or *kosoto*, ‘*they would have boiled some herbs for the baby to be drinking until it is ready to suck breast*’ (FGD, Household heads). These herbal concoctions include ‘*small quantities of the red millet, the ordinary millet, the shea nut shell and groundnut put together in water and boiled for the baby’* (FGD, Grandmothers).

"R:Some years ago when a woman delivered, they would go to another house to get another woman who had delivered not quiet long to come and feed the newly born baby until the day of pouring “sooru”. On this day, the newborn would then be breast fed by its mother."

"M:How long would it take to do all that?"

"R:If it was a boy it was three days, if it was a girl, four days for the Kassenas to pour “sooru.” After that the baby could then be breast fed by the mother.(FGD, Compound Heads)"

"Despite multiple reports of this practice, some community members suggested that women are beginning to shy away from this practice because ‘there are so many diseases with us so they (mothers) don’t allow other nursing mothers to breast feed the babies’ (FGD, Compound heads)."

Finally, data suggest that traditional healers play an important role in helping women address breastfeeding problems, both through identifying nutritional supplements for the mother as described previously and assisting in cases where infants are not feeding properly. As one household head explained in the context of an infant not feeding, it is often important to ‘*consult our soothsayers to know why it (the infant) is not feeding and make sacrifice.*’ (FGD, Household heads).Sacrifices might include the killing of a chicken or other animal as an offering to the ancestors for assistance.

### Macro-level themes

In addition to addressing tangible issues such as when and how breastfeeding is practiced in rural northern Ghana, the data collected here suggests three over-arching themes: 1) The importance of religion in influencing breastfeeding behaviors; 2) The critical role played by grandmothers; and 3) The influence of location of delivery on breastfeeding adoption and maintenance.

### The role of Religion

Religion appeared to be associated with reported compliance with breastfeeding guidelines, with noticeable differences between Christians and those who practice traditional religion. Pouring of libation (pouring a religiously significant liquid on the ground as an offering to the ancestors) and feeding the baby ritual foods were activities reported frequently among those practicing traditional religion. ‘O*n the day the baby is born, the baby will not be given anything. The compound head has to go and consult the gods before they can start to feed the baby*.’ (FGD, Compound head) Also, the ritual of *puure-nyoone* or *wobi-biisa* and *sooru o*r *kosoto* that delay initiation of breastfeeding by 3 days for male babies and 4 days for female babies was reportedly a behavior of traditional worshippers. When asking about such rituals in Christian households, the responses were typified by the following mother’s comment: ‘*We are Christians, we don’t do those things.’ (IDI, Mother of new born, 2 or more deliveries)*

### Grandmothers as Gatekeepers

The data also showed that grandmothers are extremely powerful figures in newborn health activities in this part of northern Ghana. The following exchange between an interviewer and two heads of households is particularly illustrative.

"M: Does the baby’s mother always try to feed it after it is born?"

"Household Head 1: Yes they try to breast feed it after delivery but the Grandmother … usually don’t agree."

"M: Why?"

"Household Head 1: They always say the first breast milk is bitter and not good for the baby’s consumption."

"Household Head 2: Yes that is what they say that it is not good for them since it is bitter. The mother’s always want to, but the Grandmothers don’t allow them to do so. (FGD, household heads)"

Healthcare providers similarly note the influential role of grandmothers to promote unhealthy supplementary feeding;

"(Water intake is one) habit which is a tradition which is dying a little hard. You have some of the old, you know here in this environment, the grandmothers, they have a lot of clout. When I say grandmothers, I mean, I’m talking about the mother-in-laws of the woman, the grandmother of the baby. So the mother-in-law of the woman.Their husband’s mother.They (have) a lot of power. Usually they, the married woman lives in the husband’s compound. So it’s not her own mother who matters. Her mother is living elsewhere. So it’s the husband’s mother who calls the shots. And usually they want to bring their longtime experience in… So this is what we used to do, and this is what you’ve got to do. But I think that things are beginning to change slowly. There are still some that think that the baby should be given water. Why shouldn’t the baby be given water? This is a very hot environment, the baby is thirsty. So, we still suspect that some of them may quietly be giving water to the babies. (IDI, Healthcare Provider)"

### Influence of delivery location

Data also suggest that many of the factors described vary by place of delivery. For example, one mother with a newborn infant suggested that ‘*When you deliver in the hospital, you breast feed it and continue at home,’* (IDI, Mother new born, 2 or more deliveries) whereas delivering outside the hospital may prevent women from immediately breastfeeding or maintaining exclusive breastfeeding in the face of competing advice from traditional birth attendants, grandmothers, and other community members.

The role of colostrum also varies depending upon delivery location. As described, outside the hospital, colostrum is often tested to determine breast milk’s suitability for an infant. Yet in hospital settings, ‘*these days, they make babies take colostrum at the hospital because it is thick and healthy’* (FGD, Compound heads).

## Discussion

This qualitative study among more than 250 individuals in rural northern Ghana suggests that community members are knowledgeable about the importance of breastfeeding, and most women with newborn infants do attempt to breastfeed. However, data suggests that traditional practices related to breastfeeding and infant nutrition continue, despite knowledge of clinical guidelines. Such traditional practices include feeding newborn infants water, gripe water, local herbs, or traditionally meaningful foods such as water from the flour of guinea corn(yara’na). These findings are similar to existing literature that suggests the introduction of foods other than breastmilk in the first 6 months is a common practice in sub-Saharan Africa [19,20].

In this district of Ghana, there are significant cultural traditions associated with breastfeeding – including testing the breastmilk for bitterness before allowing a woman to breastfeed. Such a test – in which an ant is dropped into breastmilk and its demise is seen as an indication of the toxicity of the milk– is performed by grandmothers and other influential community members. Given the community hierarchy, such a tradition is likely to be challenging for a young mother with a newborn to overcome. Traditionally, breastfeeding is delayed by three days for male and four days for female infants by first time mothers if a test of the mother’s milk shows that it is toxic. Also, prelacteal feeds are given or a wet nurse is used while the mother goes through the traditional process of purifying the breast. Changing such community-rooted traditions will require the engagement of the community hierarchy in breastfeeding campaigns.

Generally, there was a perception that most traditional ways of feeding infants are giving way to current recommendations. This transition is positive and appeared to be associated with religion. Individuals, who practice religions, such as Christianity, that prohibit the offering of sacrifices, consulting of sooth-sayers and performing rituals such as testing colostrum by putting ants in it, are more likely to promote and support appropriate infant feeding practices. Religious leaders could therefore, play a crucial role in shaping societal conduct towards breastfeeding.

Our data also indicate that grandmothers – typically the mother-in-laws – wield an enormous amount of power in these communities. These results are similar to other studies both in Ghana and beyond. In Eastern Ghana, Otoo et al. found that one major barrier to breastfeeding cited by women was pressure from family, specifically, the grandmother [21]. The authors report women becoming confused by the mixed messages they receive and often defaulting to adding water or other supplemental foods [21]. Similarly, a study in Mozambique found that while mothers had heard the recommendation to exclusively breastfeed, other family decision makers had not and expressed skepticism about its feasibility [22].

Infant feeding is viewed traditionally as a gender role for women [23]. However, it was evident from our study that males also play a crucial role in influencing breastfeeding behavior. In the KND, compound and household heads are the gender roles of males and these groups demonstrated good knowledge of breastfeeding recommendations. Being a patriarchal society, men generally dictate the ways of life of the people. Men prepare the herbal teas, pour the libations and consult the sooth-sayers and these activities were reported to influence breastfeeding behavior. This finding corroborates with the study by Littman et al who found that a strong approval of breastfeeding by fathers was associated with a high incidence of breastfeeding (98.1%), compared to only 26.9% breastfeeding when the father was indifferent to feeding choice (*P* < 0.001) [24].

Finally, our findings caution that conclusions about breastfeeding and supplemental feeding may be highly contingent upon where women delivered their babies. Babies delivered in a healthcare facility may be more likely to initiate breastfeeding early and maintain exclusive breastfeeding. In addition, our data suggest that women delivering in a facility may be more likely to give their babies colostrum than women delivering outside the facility. These findings are similar to those of Tawiah-Agyemang et al. (2008) [19] in the middle belt of Ghana. Negative perceptions of colostrum have been well documented elsewhere and strategies to disabuse the minds of mothers and grandmothers have been developed by Linkages, Ghana [25,26]. However, aggressive campaigns and creation of community platforms to guide mothers to breastfeed appropriately have not been vigorously pursued.

Despite its strengths, there are limitations to this study. First, interviews were conducted by undergraduate- and graduate-student interviewers. It is possible that results might have been different if the community members perceived the interviewers to be more similar to themselves. It is also possible, on the other hand, that community members were less guarded among students than they might have been with local peers. Given the volume of information readily volunteered and the 20-year history of the Navrongo Health Research Center conducting interviews in the community with interviewers very similar to those used in this study, we believe respondents were not inhibited by the student status of interviewers.

Second, this study relied upon self-reported data and does not include, for example, independent assessments of infant feeding practices. Nonetheless, the consistency of the findings and the wide variety of respondents who reported similar occurrences suggest that self-reported data in this case is valid. Finally, qualitative data were translated from the local language into English for analysis. It is possible that nuances of meaning were lost in the translation process, despite our efforts to maintain the integrity of the data by retaining local words when no English translation was sufficient.

### Implications

The results presented here have enormous implications for improving infant nutrition in rural Ghana. Perhaps most critically, our results suggest that while most women have heard and absorbed messages regarding exclusive breastfeeding, their family members may not. In a setting where decisions are often made by grandmothers and husbands and community leaders rather than individual women, this suggests that public health interventions would be well served to target the broader community, specifically grandmothers, fathers and local healers when trying to increase appropriate breastfeeding rates. There is a precedent for this: One study in Senegal successfully utilized grandmothers to encourage pregnant women to reduce their workload during pregnancy [27], while the Health Hut system also in Senegal has reported similar improvements in health care process indicators since the incorporation of grandmothers and fathers [28]. We propose a similar intervention in northern Ghana, in the realm of optimal newborn health practices, specifically nutrition.

Our results also suggest that religious leaders may be an important target in improving early onset of breastfeeding, exclusive breastfeeding, and maintenance of breastfeeding. Religious practices, especially among those who practice traditional religion, appear to favor supplemental feeding from a very early age. Integrating religious leaders into future public health interventions has the potential to significantly alter community attitudes, beliefs, and norms with regard to what is appropriate newborn nutrition.

## Conclusions

In summary, our exploratory work suggests that delayed, supplemental and alternate breastfeeding practices abound in northern Ghana, despite respondents demonstrating deep knowledge of recommended feeding practices. These practices, often imbued with great social and traditional significance may need to be re-evaluated in the light of the high neonatal and infant mortality rates in the region. Policy-makers and programmers will do well to incorporate the whole cross-section of the community in any discussion or interventional studies, especially grandmothers, fathers and religious leaders who play a pivotal role in determining newborn health practices in the home.

## Abbreviations

WHO: World Health Organization; UNICEF: United Nations Children’s Fund; KND: Kassena-Nankana District; NHRC: Navrongo Health Research Centre; NHDSS: Navrongo Health Demographic and Surveillance System; CKI: Community Key Informant; IDI: In-depth Interview; FGD: Focus Group Discussion; SMO: Senior Medical Officer; TBA: Traditional Birth Attendant.

## Competing interests

The authors declare that they have no competing interests.

## Authors’ contributions

RA, CE, CM and PA conceived the study, participated in its design, data acquisition and analysis and helped draft the manuscript. SR participated in data analysis and drafting of the manuscript. GL and GA, helped in data acquisition and analysis. JW and AHhad significant intellectual input into the analysis and drafting of the manuscript. All authors read and approved the final manuscript.

## Authors’ information

RA, MPH, Senior Health Research Officer, Navrongo Health Research Centre. CE, MD, FAAP, Clinical Assistant Professor (Pediatrics), Maternal and Child health, University of North Carolina, Chapel Hill. CM, MPH, Managing Director of Global REACH at the University of Michigan Medical School and Research Investigator in the Department of Medical Education. SR, MPH, Research Associate at Global REACH, University of Michigan Medical School. JW, MD, MPH, MD, Principal Medical Officer/Clinical Research Fellow at the Navrongo Health Research Centre, PA, PHD, MA, Senior lecturer, School of Public Health, University of Ghana. GL, BA, Research Assistant, Navrongo Health Research Centre. GA, BA, Research Assistant, Navrongo Health Research Centre. AH, PhD, MD, MPH, Director, Health Research and Development Directorate, Ministry of Health, Ghana.

## Pre-publication history

The pre-publication history for this paper can be accessed here:

http://www.biomedcentral.com/1471-2393/12/76/prepub
